# Impression of multiple implants using photogrammetry: Description of technique and case presentation

**DOI:** 10.4317/medoral.19365

**Published:** 2014-03-08

**Authors:** David Peñarrocha-Oltra, Rubén Agustín-Panadero, Leticia Bagán, Beatriz Giménez, María Peñarrocha

**Affiliations:** 1 Master of Oral Surgery and Implant Dentistry. Department of Dental Medicine, University of Valencia, Spain; 2 Associate Lecturer in Prosthetic. Department of Dental Medicine, University of Valencia, Spain; 3 Collaborating Lecturer in Oral Medicine. Department of Dental Medicine, University of Valencia, Spain; 4 Master of Prosthetics. PhD student. Department of Prosthetics, Complutense University of Madrid, Spain; 5 Full Lecturer in Oral Surgery. Department of Dental Medicine, University of Valencia, Spain

## Abstract

Aim: To describe a technique for registering the positions of multiple dental implants using a system based on photogrammetry. A case is presented in which a prosthetic treatment was performed using this technique. Study Design: Three Euroteknika® dental implants were placed to rehabilitate a 55-year-old male patient with right posterior maxillary edentulism. Three months later, the positions of the implants were registered using a photogrammetry-based stereo-camera (PICcamera®). After processing patient and implant data, special abutments (PICabutment®) were screwed onto each implant. The PICcamera® was then used to capture images of the implant positions, automatically taking 150 images in less than 60 seconds. From this information a file was obtained describing the relative positions – angles and distances – of each implant in vector form. Information regarding the soft tissues was obtained from an alginate impression that was cast in plaster and scanned. A Cr-Co structure was obtained using CAD/CAM, and its passive fit was verified in the patient’s mouth using the Sheffield test and the screw resistance test. Results and Conclusions: Twelve months after loading, peri-implant tissues were healthy and no marginal bone loss was observed. The clinical application of this new system using photogrammetry to record the position of multiple dental implants facilitated the rehabilitation of a patient with posterior maxillary edentulism by means of a prosthesis with optimal fit. The prosthetic process was accurate, fast, simple to apply and comfortable for the patient.

** Key words:**Dental implants, photogrammetry, dental impression technique, CAD/CAM.

## Introduction

Dental implants are one of the most widely used therapies for the rehabilitation of partially or completely edentulous patients. It is scientifically proven that achieving proper passive fit of the implant-supported prosthesis improves the long-term prognosis of this therapy (1-5).

The classic system for fabricating implant-supported prostheses involves taking impressions, and after placement of the implant analogues, subsequent casting in plasterto makeimpression transfers. In order to achievean adequate passive fit of the prosthesis, the first step must be to obtain a correct registration of the three-dimensional position of the implants (6).

Conventional impression techniques use abutments that, screwed onto the implants’ prosthetic platforms and encompassed by setting material, should register and transfer the spatial position of the implant. These methods involve time-consuming clinical work and the use of impression materials and techniques that often fail to achieve a perfectly accurate master cast. Moreover, these techniques are generally unpleasant for the patient (7,8).

The literature reflects the increasing application of digital techniques at different stages of dental implant therapy (9). At the stage when impressions are taken, intraoral scanners are being introduced into clinical practice. The technique avoids the need for registering implant positions with impression materials and plaster modelsand so avoids the slight dimensional distortions that these materials can cause and ensures precision when it comes to reproducing intraoral dimensions (7,10-12).

These instruments are a promising alternative for obtaining direct intraoral impressions in a fast and comfortable way for the patient. However, they are not indicated for implant rehabilitations requiring more than 3-4 pieces.

Photogrammetry is a novel option for reliable, direct intra-oral registration of the positions of multiple implants. It is a techniquefor determining the geometrical properties of objects and their spatial arrangement from photographic images. Its most important feature is the precision with which it can measure objects without direct contact.

Photogrammetry is useful in many sciences and fields. It has been applied mainly to topography, but there are many non-topographic applications, including different areas of medicine such as radiology (to improve accuracy), surgery (neurosurgery, plastic surgery, sinus surgery) or rehabilitation (13,14).

In dentistry, this technique has been used to study the shapes and positions of teeth, dental arches and maxillary and mandibular bones. In orthodontics, it allows the three-dimensional analysis of the variations of the palate while performing rapid palatal expansion techniques and evaluating the achieved dental movement (15-18). Recently, its application in dental implant surgery planning has also been reported (19).

In the field of implant dentistry, it has been used to check the accuracy of other impression techniques, by analyzing the differences between models obtained using different techniques and materials (20). As long ago as 1999, Jemt and Bäck (21) proposed photogrammetry as an alternative to conventional impression taking but since then no development of this application has been reported.

The most important quality of this technology - measurement accuracy - is the key to success in implant impressions. Therefore, its application may be a very useful technique that will improve dental implant therapy.

The aim of this report is to describe this technique applied to record the position of multiple dental implants using a system based on photogrammetry. A case is presented in which a prosthetic treatment was performed successfully using this technique.

## PICcamera®

The *PICcamera®* (PICdental, Madrid, Spain) is a stereo-camera that records implant positions in the mouth by means of photogrammetry. It comprises two CCD cameras specially designed and optimized for clinical use, which accurately determine the position of the implants by means of the identification of abutments screwed on implants with unique individual coding (*PICabutment®*, PICdental).

The camera has an infrared flash that constantly illuminates the scanned object while eliminating the shadows that occur with ambient light. The *PICcamera®* needs to capture 50 three-dimensional photographs for every two *PICabutment®*. To do this, it automatically takes ten extraoral pictures per second with an error of less than 10 microns. The registered angles and distances between implants are interrelated and treated as a unit.

System software calculates average angles and distances between implants from these photographs, obtaining an accurate relative position of each implant in vector format. This is the *PICfile®* (PIC Dental), which contains all the information on implant positions, geometries, connections, healing abutments and screws that are later requiredby CAD/CAM software.

## Clinical Procedure

**Figure 1:**
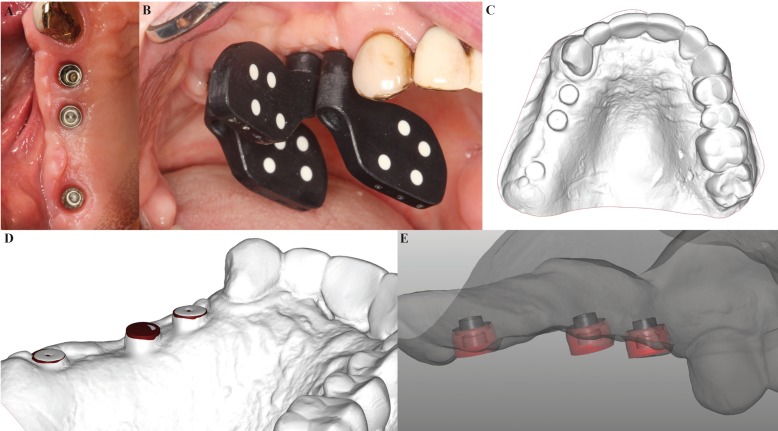
A) View at three months after the placement of three implants in the first quadrant; B) Attachments (PICabutment®) with unique individual coding screwed onto implants; C) Digitized plaster model; D) Alignment by means ofBest-fit®from the PICfile® vector file and digitized plaster model; E) Relative interface positions of the future prosthesis in relation to the gums.

A 55-year old male with no relevant medical history came to the Oral Surgery Unit of the University of Valencia requesting the rehabilitation of hisedentulous right maxillary posterior region with dental implants. After checking the presence of enough residual alveolar bone height by means of a panoramic radiograph, three *Euroteknika®* (Euroteknika Iberia, Barcelona, Spain) implants were placed of 4.1 mm in diameter (Fig. 1).

Three months later, the position of the implants was registered using the *PICcamera®* (PICdental).

Firstly, the patient’s demographic and medical data were entered into the system. Then, the positions and the references of the implants (manufacturer, model, platform diameter, diameter and height of the healing abutments), and the code of each *PICabutment®* were introduced. The *PICabutments®* were screwed onto each implant (Fig. 1), and the *PICcamera®* was placed 15-30 cm away from the patient’s mouth with a maximum angle of 45º with respect to the *PICabutments®*. Once the camera had detected that the position was correct, it automatically captured 50 three-dimensional photographs foreach two attachments. For this clinical case, 150 pictures were taken in less than 60 seconds to obtain the relative position of each implant (angle and distance) in vector format. This information was automatically compiled into a vector *PICfile®* (PIC-dental).

The healing abutments were placed and an alginate impression was taken and cast in plaster. The plaster model was scanned with a 3D scanner in open STL format to obtain information regarding the patient’s soft tissues (Fig. 1). This information was then introduced in the CAD software together with the *PICfile®*.

The *PICfile®* and the digitized plaster model were aligned with the *Exocad®* software (Exocad GmbH, Darmstadt, Germany) using three-point registration and subsequently improved alignment by *Best-fit®* (Fig. 1). This process transferred the relative position between implants to the digital model which provided the shape of the soft tissues, thus leaving the interfaces of the future prosthesis in relation to the patient’s gingiva (Fig. 1).

**Figure 2:**
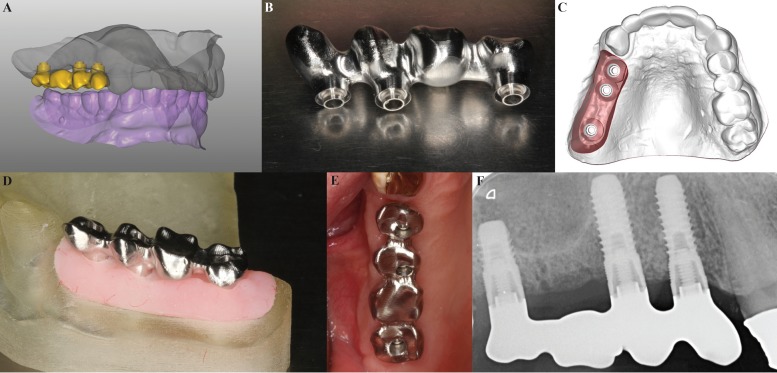
A) Upper and lower plaster models and design of the prosthetic structure; B) Machined metal structure in Cr-Co; C) Digital working model; D) Sterolithography working model with false gums; E) Checking the metal structure in the mouth; F) Periapical radiograph during the Sheffield test.

A model of the antagonist arch was also scanned,entered in the CAD software to provide occlusal references, and the prosthetic structure was designed using *Exocad®* (Exocad, GmbH) in STL format (Fig. 2). The design was sent to be machined in chrome-cobalt (Cr-Co) by a five-axis milling machine (Fig. 2).

To build a working model, the digital model was processed providing the specific geometries of the implant connections (Fig. 2) and it was manufactured by means of stereolithography using a 3D printer (Objet 250® Eden, Israel). The model was processed in a manner that allowed the addition of false gum for further work in the laboratory (Fig. 2).

Once the internal structure of the implant-supported fixed partial denture had been fabricated, its passive fit was checked in the patient’s mouth. The Sheffield and one-screw tests were used: a distal screw was placed–with the screw at 14 in this case - and a periapical radiograph was obtained to check the correct prosthetic settlement on the other two implant connections (Fig. 2). The screw resistance technique was used as a subjective complementary test of the passive fit. Distal screws (at 14 and 17) were screwed with a torque of 10 Ncm and then a medial screw was introduced verifying that the tactile sensation was soft and presented no resistance to screwing. After these verifications, the Cr-Co structure was sent to the laboratory to have the ceramic loaded.

**Figure 3:**
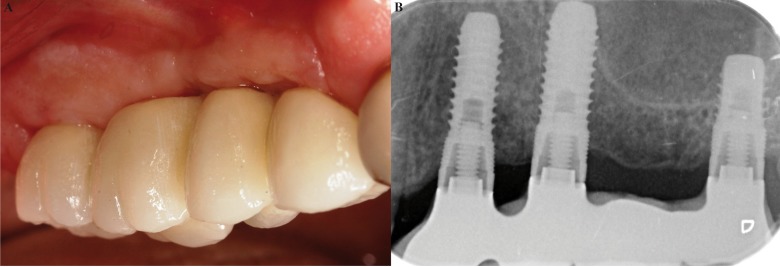
A) Placement of the finished prosthesis; B) Radiographic check-up after 12 months.

The prosthesis,once finished, was screwed onto the implants (Fig. 3), with 25 Ncm torque. Occlusal adjustments were performed and the correct settlement on the implant connections was verified with a radiograph (Fig. 3). A follow-up plan was established and twelve months after loading, the peri-implant tissues were healthy and no peri-implant marginal bone loss was observed (Fig. 3).

## Discussion

The provision of tension-free connections between implants and the prosthetic structures they support is a requirement for the medium- and long-term success of implant-supported rehabilitations. This situation can only be achieved by carrying out a prosthodontic treatment with good passive fit. Passive fit depends on all the clinical and laboratory procedures involved in fabricating the prosthesis being performed precisely and accurately, keeping the margins of error and inexactitude of each step in the process to a minimum (1,22).

*In vitro* studies have shown that discrepancies in the super-structure will be the cause of stress on the implant-supported prosthesis and subsequent failure. As long ago as 1986, Balshi described mechanical failures which he associated with laboratory work carried out using imprecise working models. Jemt *et al.* (8) and Rubenstein *et al.* (23) suggested that the fit between prosthesis and abutment is a key parameter for avoiding overloading of the fixing screw which leads to prosthetic failure.

For this reason, the taking of impressions is a fundamental step for obtaining structures with a good passive fit. There is some controversy in the literature as to which impression technique is the most reliable. Bearing in mind that with conventional techniques it is impossible to achieve a perfect passive fit, Lee *et al.* (6), in a literature review of the precision of impression techniques, found that 35% of the tests performed considered the open tray technique to be the most precise, 15% the repositioning technique and 50% found no statistically significant differences between the two. As for the number of implants in relation to precision, with three or less implants there did not appear to any difference between techniques, while with four or more the open tray technique was found to be recommendable (6). The greater accuracy of the open tray technique is corroborated by Del´Acqua MA *et al.* (24), who studied average discrepancy with each type of impression coping, this being 116.97 µm for repositioning copings and 57.84 µm for open tray copings.

The concept of photogrammetry consists of ‘metering what is written in light,’ in other words, obtaining reliable metric information from photographs.The photogrammetry method extends the two-dimensional information provided by photos into three dimensions; using various cameras, the shape of each of the photographic objects and their location in space are reconstructed in relation to an external system of reference points. To make the necessary calculations for reconstruction, special cameras are required that are able to identify this system of reference points.

Photogrammetry has been applied in various areas of medicine (13,14) and dentistry (15-19). In implant dentistry, it has been used in vitro research to test the reliability of other impression techniques (20). As early as 1999, Jemt and Bäck (21) described its use for registering the positions of dental implants intraorally. They compared this technique with conventional impression taking, concluding that photogrammetry offered a valid alternative. Since then the technical advances have been considerable but have not been accompanied by any development of the application of photogrammetry for the purposes of implant dentistry. The present article presents this new system for registering, simply and precisely, the positions of multiple dental implants.

Photogrammetry allows the registering of the exact three-dimensional locations of the implants, transferring all the information required to fabricate the prosthesis directly from the patient’s mouth to a computer file. The technique avoids the inconvenience accompanying conventional impression techniques. There is no need for impression abutments, implant body analogues, trays and impression materials. The *PICcamera* measures angles and distances between prosthetic attachments placed on the implants, allowing the patient total freedom of movement and the presence of blood, saliva or any other organic or inorganic residue does not affect measurement precision. Avoiding so many procedures and materials reduces the possibility of error, saves time – both the number of visits to the clinic and their duration – economic cost and patient discomfort in comparison with conventional impression taking procedures.

Photographic and video scanners share some of the advantages of photogrammetry. Scanners generate 3D images on the basis of a cloud of points that are able to reproduce surfaces. To join the points they use an algorithm called *Best-fit®*, which make as many points as possible coincide. Although practical evidence is limited, theoretically these successive unions of clouds of points could cause an accumulation of error. For this reason, reliability diminishes progressively according to the increasing number of implants analyzed (25). But in contrast with intraoral video and photographic scanners, photogrammetry generates director vectors of the exact position of the implants in relation to one another. The information that makes it possible to calculate the positions of the implants is obtained without superimposing photos, which potentially provides greater precision and a better prosthetic fit.

With the implant positioning determined by the *PICcamera®*, and an alginate impression taken of the soft tissues, the laboratory can fabricate the prosthetic structure using CAD/CAM, without the need for casting attachments or milling (26). In addition, the technique described in the present article does not require any impression materials or cast models, which inevitably undergo dimensional changes that will reduce the precision of the prosthesis (27). In this way, the combination of registering implant positions by photogrammetry and fabrication by CAD/CAM can potentially reduce the risk of errors occurring during the production of the prosthetic structure.

The clinical evaluation of passive fit between implants and prosthetic structures is difficult and not very objective. Diverse methods for checking fit have been suggested, but none has been established as a standard test. In the present case, the Sheffield test and the one-screw resistance test were used to check fit. The Sheffield test has been shown to be an efficient clinical test of passive fit, especially in cases with multiple implants and extensive prosthetics. The screw resistance test has the disadvantage of introducing subjectivity into the evaluation, but is considered a precise way of detecting discrepancies (28).

Registering implant positions with the *PICcamera* improves patient comfort in comparison with conventional impression taking techniques. The technique avoids the introduction of impression materials which must be kept in place in the mouth for an average setting time of 5-8 minutes and can provoke nausea and discomfort. Furthermore, the photogrammetry procedure can be interrupted if necessary and taken up again later on.

The clinical application of this novel photogrammetry system for registering the positions of multiple implants allowed the rehabilitation of a patient with extreme maxillary free end edentulism with a prosthesis of optimal fit. The prosthetic fabrication process was precise, fast, simple for the dentist and comfortable for the patient.
